# Conventional versus helical blade screw insertion following the removal of the femoral head screw: a biomechanical evaluation using trochanteric gamma 3 locking nail versus PFN antirotation

**DOI:** 10.1186/s12891-021-04658-y

**Published:** 2021-09-08

**Authors:** Hong Man Cho, Kwang Min Park, Tae Gon Jung, Ji Yeon Park, Young Lee

**Affiliations:** 1 Department of Orthopedic Surgery, Gwangju Veterans Hospital, 99 Cheomdanwolbong-ro, Gwangsan-gu, Gwangju, 62284 South Korea; 2 Osong Medical Innovation Foundation, Medical Device Development Center, Cheongju, South Korea; 3 Veterans Medical Research Institute, Veterans Health Service Medical Center, Seoul, South Korea

**Keywords:** Conventional blade, Helical blade type, Hip screw, Biomechanical study, Pullout strength

## Abstract

**Objective:**

When a hip screw needs to be changed, choosing between the conventional (C-type) and helical blade (H-type) types is difficult. In this biomechanical study, we compared these two screw types relative to the type of the initial screw used.

**Methods:**

C- or H-type screws were inserted (leading screw) in three types of polyurethane bone models (Sawbone, Pacific Research Laboratories, Inc., Washington, USA: 130 × 180 × 40 mm) of different bone mineral densities (pounds per cubic feet [PCF] 5, 80 kg/m^3^; PCF 10, 160 kg/m^3^; and PCF 15, 240 kg/m^3^), and then successively or alternately inserted (following screw) after the leading screw removal. An original model (original C and H) of a leading screw without removal was created as a control. The strengths of resistance to pullout (PO) and rotational stress were measured. For each experimental condition, there were 30 experimental models.

**Results:**

The original C screw was superior in PO strength, and the original H-type screw was superior in rotational strength. When the C- or H-type screw was the leading screw, using the C-type screw again as the following screw (C1-C2, H1-C2) showed the greatest resistance to PO, and using the H-type screw as the following screw (C1-H2, H1-H2) showed superior resistance to rotational strength. However, the rotational strength of the C2 screw decreased by more than 50% compared with that of the original C screw. Moreover, the PO and rotational strengths of the H2 screw decreased to less than 30% overall compared with those of the original H screw.

**Conclusion:**

The H-type screw should be used for second-time screw insertion procedures in cases where it is difficult to choose between PO and rotational strengths.

## Background

Femoral intertrochanteric fractures (IT Fx) are representative osteoporotic fractures in elderly patients, and their incidence has markedly increased in aging societies [1]. For most IT Fx, the complications are prevented with early surgical management. Surgical treatment using the nail system for hip (NSH) is emerging as a popular procedure [2]. The representative types of hip screws inserted into the femoral head in the NSH system are the conventional-type (C-type) and helical blade-type (H-type) screw; the most stable insertion site is the center-center of the femoral head [3]. Although the NSH system has been successfully used for treating IT Fx [4, 5], the overall rate of mechanical complications was reportedly 8% [6] in Gamma nail and 10.8% [7] in PFNA; thus, revisions may be required for to prevent fixation failure or trauma. Bojan et al. [8] reported that fixation failure following intramedullary fixation occurred in patients with at least one of the major factors: fracture classification, inadequate reduction, and improper positioning of the delay screw. For revisional surgery, osteosynthesis is reattempted using various implants for internal fixation or arthroplasty. Whereas hip replacement after intertrochanteric fixation failure was usually salvaged where lag screw cut-out generally occurs with little proximal bone remaining, or in cases of severe osteoporosis. Furthermore, osteosynthesis revisional surgery through open reduction and internal fixation preserves the native femoral head; plates with 95° blades are useful when they target bone in the inferior portion of femoral heads that have not been violated by prior fixation devices [9]. In specific conditions, such as when the femoral head bone defect is small and the bone quality is adequate for reinserting the hip screw, a method of attempting osteosynthesis is by replacing the NSH system currently in place while replacing the length of the intramedullary nail. The success rate of intertrochanteric fixation failure after refixation was approximately 66.7% [10]. At this time, if the position of the previously inserted hip screw (leading screw, number 1) is center-center, the new hip screw (following screw, number 2) is reinserted in the same position as the leading screw. Surgeons must select a representative screw type (C- or H-type). The concern is whether the following inserted screw should be of the same type as the previously inserted leading screw or of another type. This is because the most stable fixation must be obtained to reduce the risk of failure after reoperation. Therefore, in this biomechanical study, we attempted to determine the best screw type to use in second-time insertions in relation to the original type, by comparing C (trochanteric gamma 3 locking nail) and H (PFN antirotation) type screws in a polyurethane synthetic bone model.

## Methods

### Experimental design

**Fig. 1:**
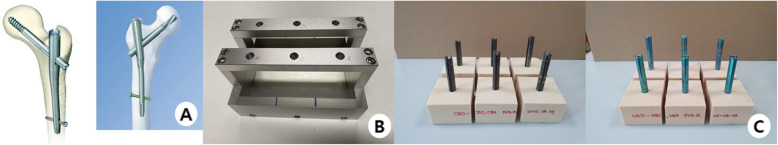
**A** The conventional- and helical blade-type hip screws used. Cross and longitudinal sections after insertion into the bone model are indicated. **B** A special box made to insert a screw at the same location and depth in the polyurethane bone model for testing. **C** Two types of hip screws are inserted in the experimental bone model, which are cut to the same size and height

This study used an experimental polyurethane-based artificial bone model (Sawbone, Pacific Research Laboratories, Inc., Washington, USA: 130 × 180 × 40 mm) which is widely used in biomechanical experiments. IT Fx occur not only in patients with osteoporosis but also in patients with an osteopenic bone mineral density (BMD). Thus, the experimental bone models reflected three types of BMD: severe osteoporotic (pounds per cubic feet [PCF] 5, 80 kg/m^3^), mild osteopenic (PCF 10, 160 kg/m^3^), and osteopenic (PCF 15, 240 kg/m^3^). Two types of hip screws were used: a third-generation gamma nail (trochanteric gamma 3 locking nail; Stryker, Germany) as the C-type screw and a PFN antirotation (PFNA-II, Synthes, Solothurn, Switzerland) as the H-type screw. The length of the hip screws were uniform at 100 mm (Fig. 1A). A 130 × 180-mm bone model was uniformly divided into six equal 60 × 60-mm parts. Thereafter, 60 × 60 × 40-mm (width–length–height) lab blocks were prepared. A total of 2160 lab blocks (720 per BMD model: PCF 5, 10, and 15) were produced. A special device was designed to ensure that the femoral head screw was inserted at the same position and depth for each block (Fig. 1B).

The following experimental designs were applied: (1) insertion of the original screw (original C and original H) as the leading screw, and without removal, (2) removal of the leading screw and insertion of a subsequent screw of the same type without reaming (C1-C2, H1-H2), (3) removal of the leading screw and insertion of a following screw of a different type (C1-H2, H1-C2) after reaming (RO), and (4) removal of the leading screw and insertion of a subsequent screw of a different type (C1-H2, H1-C2) without reaming (RX). By applying these four experimental conditions to the two different types of hip screws, eight experimental models were obtained: original H, H1-H2, H1-ROC2, and H1-RXC2; and original C, C1-C2, C1-ROH2, and C1-RXH2. For all eight models, the pullout (PO), clockwise rotation (CWR), and counterclockwise rotation (CCWR) strengths of the following and original screws were evaluated, considering the BMD. We further evaluated the change in strength reduction in the PO, CWR, and CCWR strengths, between the original and the following screw (original C vs. C2, original H vs. H2).

A servohydraulic testing machine (MTS, 858 MiniBionix, Erie, USA) was used to ensure that the same force was applied during insertion, removal, and reinsertion of the hip screws. Using an automated torque tester (Vortex-i, Mecmesin Co., West Sussex, UK), an axial loading of 9.8 (1-kgf) to 29.4 (3-kgf) N at a speed of 3 r/min was applied. The entire experimental process was supervised by one orthopedic surgeon with > 10 years’ experience in surgically treating hip fractures to reduce the difference in force applied to screw insertion and removal (Fig. 1C).

The sample size was calculated using G*Power 3.1 software (Faul, Erdfelder, Buchner & Lang) for using a two-sided two sample t-test with an effect size of 0.8. Twenty-six samples were needed to achieve a power of 80% at 0.05 significance level. Owing to predicted test failures, we proceeded to 30 per each experimental model.

**Fig. 2:**
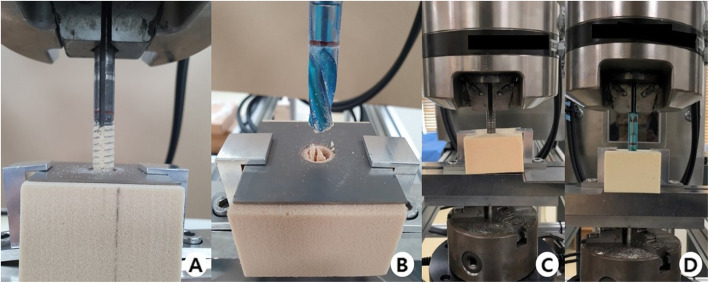
**A**, **B** Experiment for assessing the pullout strength of conventional- and helical blade-type hip screws. **C**, **D** Experiment for assessing the rotational strength of conventional- and helical blade-type hip screws

The screws were evaluated for resistance to PO, CWR, and CCWR. Evaluations were performed using 30 lab blocks for each experimental model. Each lab block was fixed firmly with a custom-made jig in the frame of a universal material testing machine (MTS Systems Corp., Eden Prairie, MN, USA). Lag in the lab block was tested according to the international test standard, ASTM F543. PO stress was applied at a speed of 5 mm/min until the screw came out (Fig. 2A, B). After deriving the load-displacement graph for the tensile loading, the maximal tensile loading force was recorded in Newton (N). The load conditions were set by referring to the bone screw pullout test method of ASTM F543; the load-displacement graph was derived using the load data recorded according to the displacement control; and N, the highest value in the graph, was called the maximum load. A rotation experiment was performed using the same experimental equipment used in the PO experiment. When inserting the hip screw into the femoral head, the rotation direction of the screw is different to the anterior and posterior of the hip joint, depending on whether the left or right hip joint was being operated on. Mohan et al. [11] reported that the rotation direction may influence the stability of the hip screw because it is different from that of the rear. Therefore, the rotation direction was divided into CW and CCW directions. Rotation was carried out at a test speed of 120°/min (Fig. 2C, D). After deriving the torque-angle graph, the maximum torque value was recorded in N-cm.

### Statistical analysis

Descriptive data are presented as means and standard deviations. Comparisons between two groups were performed using an independent t-test when normality was satisfied, and the Mann–Whitney U test when normality was not satisfied. Normality tests were performed using the Shapiro–Wilk test. For comparisons among three groups, we used analysis of variance (ANOVA) with Tukey’s test or the Games–Howell post hoc test when normality was satisfied. The Kruskal–Wallis and Dunn tests when normality was not satisfied. If the results of each ANOVA and Kruskal–Wallis test were significant, the corresponding post hoc analysis was used. Statistical significance was defined as a *p* value < 0.05. All analyses were performed using R 4.0.1 (R Development Core Team; R Foundation for Statistical Computing, Vienna, Austria).

## Results

### PO strength of the following screw after removing the leading screw

**Fig. 3:**
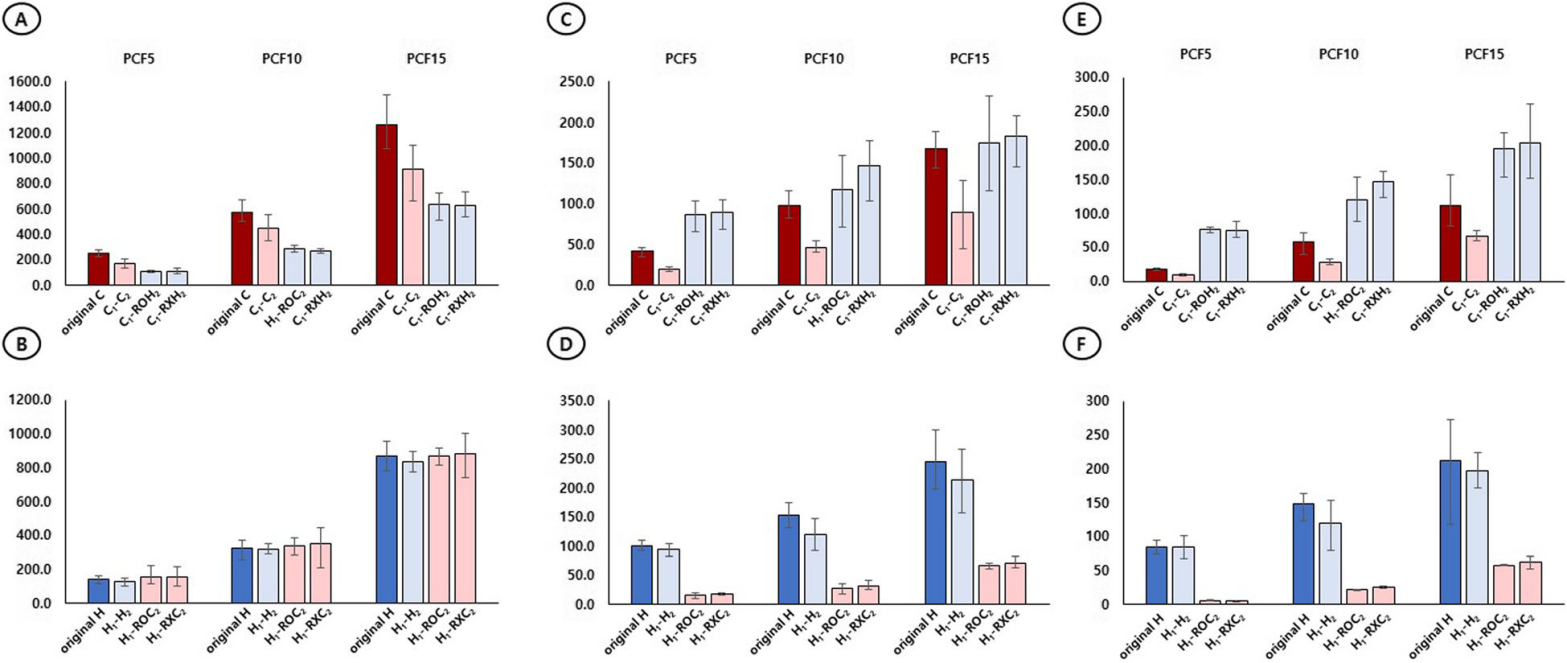
**A**, **B** The resistance strength of the following screw against pullout stress is superior to the case of using a C-type screw as the following screw (C2) in all PCFs regardless of the type of leading screw and whether reaming is performed. **C**, **D** The resistance strength of the following screw against clockwise rotation stress is superior to the case (H2) using the H-type screw as the following screw, in all PCFs regardless of the type of the leading screw and whether reaming is performed. (E, F) The resistance strength of the following screw against counter-clockwise rotation stress is superior to the case of using an H-type screw as the following screw (H2) in all PCFs regardless of the type of leading screw and whether reaming is performed

When the C-type screw was the leading screw, using the C-type screw as the following screw (C1-C2) gave the greatest resistance to PO with a significant difference compared with using the H screw as the following screw (C1-H2). When the H-type screw was the leading screw, using the C-type screw as the following screw (H1-C2) gave the greatest resistance to PO with a significant difference, compared to the H screw (H1-H2) in all bone models. When the leading screw and the following screw were different (C1-H2 or H1-C2), reaming did not affect the PO strength, except in the case of C1-RXH2, where the PO strength was significantly lower than PCF 10 (Table 1) (Fig. 3A, B).

**Table 1:** Changes in pullout strength by inserting a conventional- or helical blade-type hip screw into a polyurethane bone model of different bone densities, with the types of the leading and following screws kept the same or alternated

BMD	1	2	Pullout	*p* value
			Average (min–max) (N)	
5	C1	C2	174.55 (138–207)^a^	
5	C1	ROH2	107.03 (96–120)^b^	< 0.001
5	C1	RXH2	111.56 (90–135)^b^	
5	H1	H2	128.85 (106–148)^b^	
5	H1	ROC2	158.97 (135–194)^a^	< 0.001
5	H1	RXC2	160.36 (101–222)^a^	
10	C1	C2	445.64 (348–552)^a^	
10	C1	ROH2	285.17 (256–314)^b^	< 0.001
10	C1	RXH2	272.63 (253–290)^c^	
10	H1	H2	318.17 (279–382)^b^	
10	H1	ROC2	338.81 (284–382)^a^	< 0.001
10	H1	RXC2	351.22 (299–395)^a^	
15	C1	C2	914.53 (665–1106)^a^	
15	C1	ROH2	636.36 (507–726)^b^	< 0.001
15	C1	RXH2	626.09 (536–733)^b^	
15	H1	H2	833.24 (777–892)^b^	
15	H1	ROC2	868.54 (727–966)^a^	< 0.001
15	H1	RXC2	879.78 (744–1004)^a^	

1, leading screw; 2, following screw; C, conventional-type screw; H, helical blade-type screw RO, following screw insertion after reaming; RX, following screw insertion without reaming; *BMD* bone model density

### Rotation strength of the following screw after removing the leading screw

When the C-type screw was the leading screw, using the H-type screw as the following screw (C1-H2) gave superior resistance to rotational strength with a significant difference compared to using the C-type screw (C1-C2), regardless of the rotation direction and BMD. When the H-type screw was the leading screw, using the H-type screw as the following screw (H1-H2) gave superior resistance to rotational strength with a significant difference compared to using the C-type screw (H1-C2), regardless of the rotation direction and BMD. In particular, when the leading screw was an H-type screw and a C-type screw was the following screw after reaming (H1-ROC2), the rotational strength was significantly lower than when the reaming was not performed (CW on PCF 5 and 10 and CCW on PCF 10 and 15) (Table 2) (Fig. 3C, D, E, F).

**Table 2:** Changes in rotational strength by inserting a conventional- or helical blade-type hip screw into a polyurethane bone model, with the leading and following screws kept the same or alternated

BMD	1	2	CW rotation		CCW rotation	
			Average (min–max) (N)	*p* value	Average (min–max) (N)	*p* value
5	C1	C2	20.23 (17–23)^b^		9.35 (8–11)^b^	
5	C1	ROH2	87.24 (66–103)^a^	< 0.001	76.19 (71–80)^a^	< 0.001
5	C1	RXH2	90.08 (68–105)^a^		75.66 (65–89)^a^	
5	H1	H2	93.82 (82–105)^a^		84.39 (68–102)^a^	
5	H1	ROC2	15.69 (11–21)^c^	< 0.001	6.27 (5–8)^b^	< 0.001
5	H1	RXC2	18.69 (16–20)^b^		6.07 (6–6)^b^	
10	C1	C2	46.09 (41–54)^c^		28.73 (25–33)^c^	
10	C1	ROH2	117.53 (72–160)^b^	< 0.001	120.85 (88–153)^b^	< 0.001
10	C1	RXH2	146.90 (104–178)^a^		147.6 (123–163)^a^	
10	H1	H2	120.84 (92–147)^a^		120.05 (80–153)^a^	
10	H1	ROC2	27.03 (19–35)^c^	< 0.001	22.8 (22–23)^c^	< 0.001
10	H1	RXC2	31.61 (26–42)^b^		26.34 (24–28)^b^	
15	C1	C2	89.52 (45–129)^b^		65.83 (59–75)^b^	
15	C1	ROH2	174.30 (116–232)^a^	< 0.001	195.66 (153–219)^a^	< 0.001
15	C1	RXH2	183.82 (145–208)^a^		204.12 (152–261)^a^	
15	H1	H2	214.62 (157–267)^a^		196.6 (172–224)^a^	
15	H1	ROC2	67.17 (61–71)^b^	< 0.001	58.13 (57–60)^c^	< 0.001
15	H1	RXC2	71.49 (63–82)^b^		62.97 (52–71)^b^	

1, leading screw; 2, following screw; C, conventional-type screw; *CCW* counterclockwise; *CW* clockwise; *H* helical blade-type screw; RO, following screw insertion after reaming; RX, following screw insertion without reaming; *BMD* bone model density

### PO and rotation strength differences between the two types of original screws: original C versus original H

**Fig. 4:**
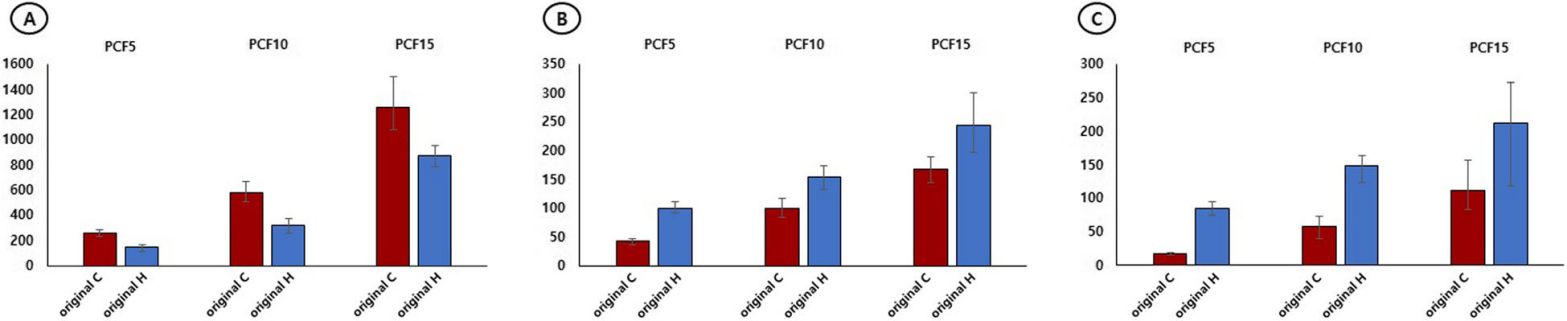
**A** Resistance strength of the C-type screw was significantly superior to that of the H-type screw against pull-out stress in all PCF (**B**, **C**) Resistance strength of the H-type screw was significantly superior to that of the C-type screw against rotation stress (either clockwise [**B**] or counter-clockwise [**C**]) for all PCF

The comparison of the resistance to PO strength between the two types of original screws (original C- vs. H-type screws) showed that the C-type screw was significantly superior to the H-type screw for all BMDs. In contrast, the H-type screw was significantly superior to the C-type screw in terms of rotational strength, regardless of the rotation direction and the BMD (Table 3) (Fig. 4A, B, C).

**Table 3:** Pullout and rotational strengths by inserting a conventional- or helical blade-type hip screw into a polyurethane bone model, with the leading (original) screw

BMD	Original C	Original H	*p* value
	Pullout		
	Average (min–max) (N)	Average (min–max) (N)	
5	253.36 (225.9–280.1)	146.15 (114.9–162.7)	< 0.001
10	575.76 (504.7–669.1)	322.81 (257.0–374.2)	< 0.001
15	1261.33 (1078.0–1497.5)	870.87 (784.1–957.7)	0.031
	CW rotation		
	Average (min–max) (N)	Average (min–max) (N)	
5	41.39 (35.5–46.1)	99.73 (91.9–110.0)	< 0.001
10	98.47 (82.8–116.6)	154.02 (132.2–174.3)	< 0.001
15	167.93 (143.4–188.8)	244.74 (197.7–300.4)	< 0.001
	CCW rotation		
	Average (min–max) (N)	Average (min–max) (N)	
5	17.68 (16.2–19.3)	85.42 (75.1–95.4)	< 0.001
10	57.68 (38.9–72.1)	147.6 (123.0–163.2)	< 0.001
15	111.27 (82.1–157.5)	211.49 (118.2–272.7)	< 0.001

*BMD* bone model density; *CCW* counterclockwise strength; *CW* clockwise strength; *C* conventional-type screw; *H* helical blade-type screw

### Changes in resistance to PO and rotational stress between the leading and following screws (original C vs. C2)

**Fig. 5:**
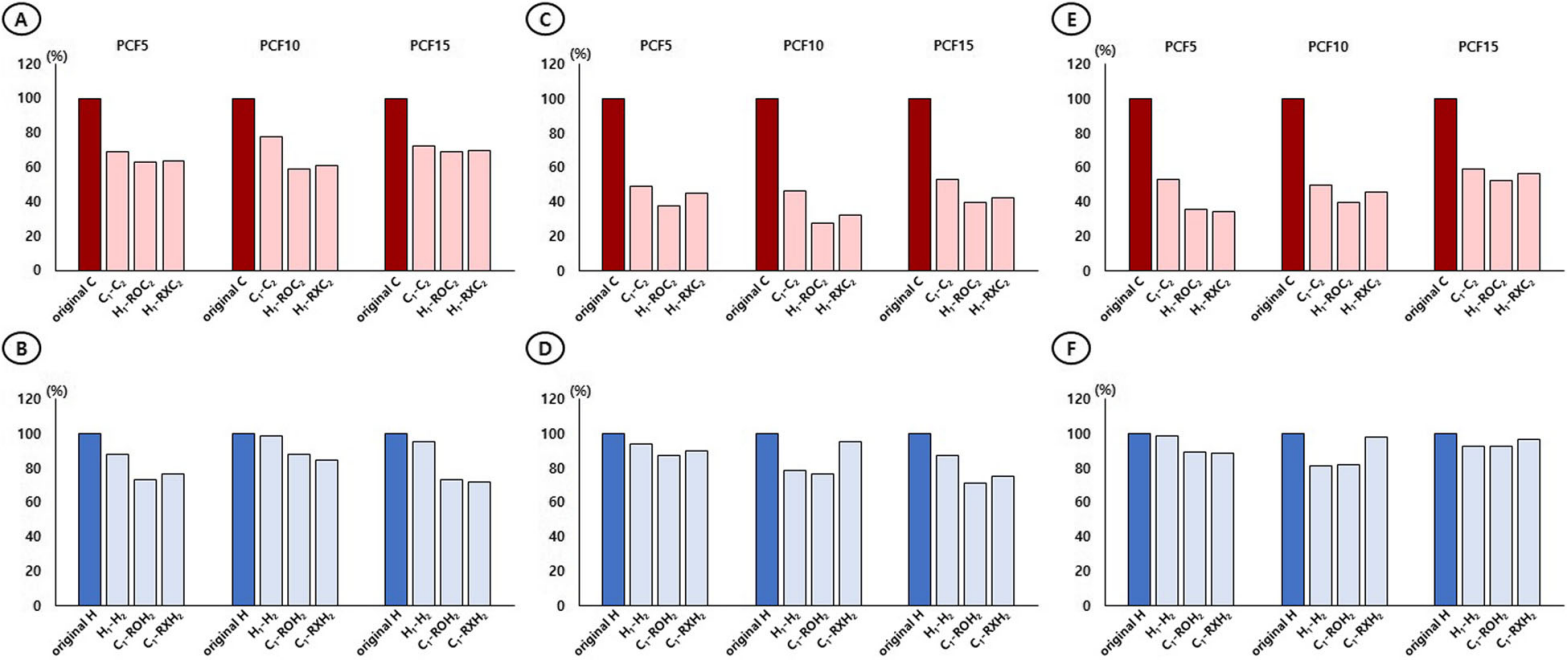
When the C-type (C2) and H-type (H2) screws used as the following screws were compared with the original screws, (**A**, **B**) the resistance strength against pullout stress decreased in C2 significantly to < 50% overall compared with that for the original C and that decreased in H2 to < 30% overall compared with that of the original H; the differences were significant except for H1-H2 (PCF 10, *p* = 0.358). **C**, **E** the rotational strength for C2 significantly decreased compared with that for the original C in both CW (**C**) and CCW (**E**). In particular, the rotation strength compared with that for the original C decreased by > 50% in all other cases except for C1-C2 (CCW, 47.13%) in PCF 5 and C1-C2 (CW, 46.69%), H1-ROC2 (CCW, 47.76%), and H1-RXC2 (CCW, 43.41%) in PCF 15. **D**, **F** The rotational strength for H2 significantly decreased compared with that of the original H in both CW (D) and CCW (F), except for H1-H2 (PCF 5, CCW) and C1-RXH2 (PCF 10 and 15, CCW). In particular, the rotational strength compared with that of the original H decreased by < 30% in all cases

When the following screw was a C-type screw (C1-C2, H1-ROC2, H1-RXC2), the PO strength for the C2 significantly decreased to < 50% overall compared with that for the original C. When the following screw was a C-type screw (C1-C2, H1-ROC2, H1-RXC2), the rotational strength for C2 significantly decreased compared with that for the original C in both CW and CCW. In particular, the rotation strength compared with that for the original C decreased by > 50% in all other cases except for C1-C2 (CCW, 47.13%) in PCF 5 and C1-C2 (CW, 46.69%), H1-ROC2 (CCW, 47.76%), and H1-RXC2 (CCW, 43.41%) in PCF 15 (Table 4) (Fig. 5A, C, E).

**Table 4:** Percent reduction of the pullout and rotational strengths of the following screws relevant to those of the original screws of the C- and H-type screws

	C2							H2					
	C1-C2		H1-ROC2		H1-RXC2			H1-H2		C1-ROH2		C1-RXH2	
	RD (%)	*p* value	RD (%)	*p* value	RD (%)	*p* value		RD (%)	*p* value	RD (%)	*p* value	RD (%)	*p* value
	Pullout							Pullout					
PCF 5	31.11	< 0.001	37.26	< 0.001	36.71	< 0.001	PCF 5	11.84	< 0.001	26.77	< 0.001	23.67	< 0.001
PCF 10	22.6	< 0.001	41.15	< 0.001	39	< 0.001	PCF 10	1.44	0.358	11.66	< 0.001	15.54	< 0.001
PCF 15	27.5	< 0.001	31.14	< 0.001	30.25	< 0.001	PCF 15	4.32	0.001	26.93	< 0.001	28.11	< 0.001
	Clockwise rotation							Clockwise rotation					
PCF 5	51.13*	< 0.001	62.10*	< 0.001	54.83*	< 0.001	PCF 5	5.93		12.52	< 0.001	9.68	< 0.001
PCF 10	53.20*	< 0.001	72.55*	< 0.001	67.90*	< 0.001	PCF 10	21.54		23.69	< 0.001	4.62	0.102
PCF 15	46.69	< 0.001	60.00*	< 0.001	57.43*	< 0.001	PCF 15	12.31		28.78	< 0.001	24.89	< 0.001
	Counterclockwise rotation							Counterclockwise rotation					
PCF 5	47.13	< 0.001	64.52^*^	< 0.001	65.69^*^	< 0.001	PCF 5	1.21	0.564	10.81	< 0.001	11.43	< 0.001
PCF 10	50.19^*^	< 0.001	60.48^*^	< 0.001	54.33^*^	< 0.001	PCF 10	18.67	< 0.001	18.13	< 0.001	1.74	0.441
PCF 15	40.84	< 0.001	47.76	< 0.001	43.41	< 0.001	PCF 15	7.04	0.015	7.48	0.015	3.48	0.218

*C* conventional-type screw; *H* helical blade-type screw; RO, following screw insertion after reaming; RX, following screw insertion without reaming; RD, strength reduction rate (%) compared with original screw; *PCF* pounds per cubic feet

*50% above strength reduction compared with original screw

### Changes in resistance to PO and rotational stress between the leading and following screws (original H vs. H2)

When the following screw was used as an H-type screw (H1-H2, C1-ROH2, C1-RXH2), the PO strength for H2 decreased to < 30% overall compared to that of the original H, and the difference was significant except for H1-H2 (PCF 10, *p* = 0.358). When the following screw was used as an H-type screw (H1-H2, C1-ROH2, C1-RXH2), the rotational strength for H2 significantly decreased compared to that of the original H in both CW and CCW, except for H1-H2 (PCF 5, CCW) and C1-RXH2 (PCF 10 and 15, CCW). In particular, the rotational strength compared to that of the original H decreased by < 30% in all cases (Table 4) (Fig. 5B, D, F).

## Discussion

Despite successful outcomes of treating IT Fx using the NSH [4, 5], revision is often required due to treatment failure, such as femoral head perforation, nonunion, loss of reduction, and periprosthetic fracture [12, 13]. Options for the revisional surgery include changing the hip screw and intramedullary nail [14], salvage procedures that augment the bone cement on the cancellous bone in the femoral head before changing the hip screw [15], and hip arthroplasty. When the revisional surgery is osteosynthesis, the preoperative evaluation of bone quality and the amount of femoral head bone loss is important because patients with IT Fx usually have low BMD, accompanied by femoral head bone loss experienced during the removal of the previously inserted screws [16]. Min et al. [10] suggested a treatment strategy for osteosynthesis in cases of minimal femoral head bone loss with a sufficient neck–shaft angle, where a 95°-angled blade plate is considered if there were no defects in the inferior part of the femoral head. Moreover, in the case of no defects in the superior part of the femoral head, a 135°-angled blade plate, dynamic hip screw, or NSH was considered. They suggested that if the superior part of the femoral head was intact, revision for osteosynthesis using a dynamic hip screw or NSH would be possible. However, they did not present the appropriate type of hip screw to be considered as the following screw based on the type of the leading screw for revisional surgery. The authors suggested that, in cases where the superior part of the femoral head is intact and the position of the leading screw is the center-center on the femoral head, it is challenging to decide the type of the subsequent screw (C- or H-type screw) for insertion in the same site as the leading screw during revision [3, 17]. Accordingly, we investigated the strength of the following screw against PO and rotational stress, considering the state of the bone defects caused by removing the leading screw.

Erhart et al. [18] reported that the PO strength is important when inserting a following screw after removing the leading screw in IT Fx revisional surgery. In our study, the C-type screw showed superior PO stress strength both when used as an original and as a following screw. Although the PO strength of H2 was smaller than that of C2, the decrease in PO strength of H2 compared with that of the original H screw was < 30%. The difference in the PO strength between C2 and H2 could be attributed to the difference in the unique design of each screw.

Rotational stability is another important factor in determining postoperative fixation failure in IT Fx [19]. Because the implants should be able to maintain the rotational stability of short and damaged proximal fragments during the fracture healing process, the choice of implant is important when a revision is performed because of failure after treatment. Here, the H-type screw was superior in terms of rotational strength, both when used as the original screw and as the following screw. Compared with the C-type screw, the H-type screw had superior rotational stability, which is likely due to lesser bone loss during insertion and removal because of its design characteristics. One study showed an elastic deformation of the trabeculae during insertion while the helical blade presses the cancellous bone [20]. Furthermore, there is an increase in bone density due to bone compactions around the H-type screw. This is a spring-back effect in which the cancellous bone of the femoral head is deformed by the rotation of the blade in the range of elasticity during the removal process. This advantage of the H-type screw was considered more prominent in cases of low BMD [21].

In our study, when the H-type screw was used as the following screw, the rotational strength decreased by ≤30%, regardless of the type of leading screw and BMD. However, when the C-type screw was used as the following screw, the rotational strength decreased by > 50% in most cases (except CCW alone on PCF 12), compared to that of the original C. In particular, in H1-ROC2 with osteoporosis (PCF 5 and 10), the rotation strength for CW and CCW significantly decreased by > 60%. Therefore, considering rotational strength, we recommend using the H-type screw as the following screw (C1-H2, H1-H2), regardless of the type of leading screw.

The differences in fixation strength when the C- and H-type screws are inserted into the cancellous bone and the stability after use for treating IT Fx have been researched. However, there is still controversy on which type of screw is superior [22–24]. In the case of inserting the following screw in the same position as the leading screw, if revision is due to fixation failure combined with excessive sliding due to the weak resistance of the screw against PO stress, the C-type screw is appropriate as the following screw, as it increases PO stress resistance. If the fixation failure is due to weak rotational stress, the H-type screw is appropriate as the following screw as it increases the rotational strength. However, since fixation failure of IT Fx initiates by rotation of the femoral head, which in turn causes excessive sliding [19, 23, 25], rotational strength should be considered more important than PO strength. In a biomechanical study by Summers et al. [13] on a C-type gamma nail and an NSH in the form of H-type screws rotated in osteoporosis and unstable fractures, it was said that because of having more bearing capacity for the load, the resistance to perforation of the femoral head was high. It would, thus, be advantageous to consider the H-type screw as the following screw if resistance to rotational stress is required before surgery, considering that just 30% less reduction in PO strength arises from the difference in design. However, in elderly patients with low BMD and accompanying bone defects, cement augmentation is necessary when reinserting the hip screw [26]. Alexander et al. [27] reported that even if the hip screw is reinserted in a good position, if there is no cement augmentation, the risk of fixation failure is high. Therefore, the femoral head bone defect should be carefully evaluated before revision.

This study has several limitations. First, we used polyurethane bone models, which do not reflect cancellous bone impaction or autogenous cancellous bone grafting that occurs with normal bone. However, using a fresh bone model would have been inappropriate to reflect various BMDs, and to consistently reproduce the experiments under the same conditions. Our study aimed not to examine the stability of fixation for IT Fx, but to determine the fixation of the following screw in the cancellous bone after removing the leading screw. Therefore, a cancellous bone model with the same shape and BMD was more appropriate than a fourth-generation compact material or a fresh femoral bone model. Moreover, the authors did not attempt a finite element model test (FEM) as it was not possible to implement reaming and reinsertion after screw removal, and it was difficult to confirm the difference in mechanical characteristics according to the thread of the screw. Second, the study did not reflect the type of IT Fx, which may affect the stability of the hip screw, and did not reflect the time elapsed between inserting the leading and following screws, which may affect the strength of the cancellous bone. In addition to the motions of the fracture site, the loss of the lateral wall of the trochanter can affect the stability of the following screw. In particular, a comparative study on bone defects in the lateral wall of the femur after removal of PFNA-II and gamma 3 showed that gamma 3 using a C-type screw as a hip screw resulted in significantly more bone defects [28]. Therefore, large-scale prospective clinical studies are necessary. Third, this study only focused on two specific types of screws while there are various other types of hip screws that are appropriate for PO and resistance against rotation in the femoral head [29]. There are several studies experimenting with variable tools, such as FEM under variable situations like temperature and force, during screw insertion. Recently, NHS, which can use both the C-type and H-type screws, has been developed and used; thus, the screw inserted into the femur head can be exchanged without replacing the nail [30]. As for the biomechanical perspective [31], bone cement augmentation significantly increases the cut-out resistance of instrumented PFNA head elements and is a valid supplementary treatment option to such nailing procedures in bones of poor quality. Therefore, a large-scale prospective study is also necessary to evaluate additional screw types. Lastly, the study design used the screw in isolation without an intramedullary nail component. It would be possible to obtain more useful information if the whole construct including the nail was used and a fracture was applied to the human femur.

## Conclusions

In conclusion, it would be more appropriate to select the C-type screw if the PO strength of the following screw is important and select the H-type screw if rotational strength is important. However, considering the differences in the unique design of the two screws and the important role of rotational strength, we believe that the H-type screw should be chosen as the subsequent screw, in cases in which it is difficult to choose between the PO and rotational strengths. Finally, the authors believe that the results of this study will act as an important reference for selecting reinsertion hip screws, specifically for patients whose screws need to be reinserted in the same position after surgical treatment with NSH.

## Data Availability

The datasets during and/or analysed during the current study available from the corresponding author on reasonable request.

## Abbreviations

IT Fx: Intertrochanteric fractures
NSH: Nail system for hip
C-type: Conventional-type
H-type: Helical blade-type
BMD: Bone mineral density
PCF: Pounds per cubic feet
RO: Reaming
RX: Without reaming
PO: Pullout
CWR: Clockwise rotation
CCWR: Counterclockwise rotation
N: Newton
ANOVA: Analysis of variance

## References

[1] Lindskog DM, Baumgaertner MR (2004). Unstable intertrochanteric hip fractures in the elderly. J Am Acad Orthop Surg.

[2] Mattisson L, Bojan A, Enocson A (2018). Epidemiology, treatment and mortality of trochanteric and subtrochanteric hip fractures: data from the Swedish fracture register. BMC Musculoskelet Disord.

[3] Kuzyk PR, Zdero R, Shah S, Olsen M, Waddell JP, Schemitsch EH (2012). Femoral head lag screw position for cephalomedullary nails: a biomechanical analysis. J Orthop Trauma.

[4] Yoo J, Kim S, Jung H, Hwang J (2019). Clinical outcomes of U-blade gamma3 nails used to treat patients with trochanteric fractures: retrospective multicenter study. Hip Pelvis..

[5] Choo SK, Oh HK, Woo SJ (2012). Mid-term results of patients with femoral intertrochanteric fractures treated with proximal femoral nail antirotation. Hip Pelvis.

[6] Hesse B, Gächter A (2004). Complications following the treatment of trochanteric fractures with the gamma nail. Arch Orthop Trauma Surg.

[7] Zhang W, Antony Xavier RP, Decruz J, Chen YD, Park DH (2021). Risk factors for mechanical failure of intertrochanteric fractures after fixation with proximal femoral nail antirotation (PFNA II): a study in a southeast Asian population. Arch Orthop Trauma Surg.

[8] Bojan A, Taglang G, Beimel C, Jonsson A, Schnettler R (2004). A retrospective analysis of cut out complication in 3066 patients treated with gamma nails. J Orthop Trauma.

[9] Petrie J, Sassoon A, Haidukewych GJ (2013). When femoral fracture fixation fails: salvage options. Bone Joint J.

[10] Min BW, Lee KJ, Oh JK, Cho CH, Cho JW, Kim BS (2019). The treatment strategies for failed fixation of intertrochanteric fractures. Injury..

[11] Mohan R, Karthikeyan R, Sonanis SV (2000). Dynamic hip screw: does side make a difference? Effects of clockwise torque on right and left DHS. Injury..

[12] Appelt A, Suhm N, Baier M, Meeder P (2007). Complications after intramedullary stabilization of proximal femur fractures: a retrospective analysis of 178 patients. Eur J Trauma Emerg Surg.

[13] Sommers MB, Roth C, Hall H, Kam BC, Ehmke LW, Krieg JC (2004). A laboratory model to evaluate cutout resistance of implants for pertrochanteric fracture fixation. J Orthop Trauma.

[14] Scola A, Gebhard F, Dehner C, Roderer G (2014). The PFNA® augmented in revision surgery of proximal femur fractures. Open Orthop J.

[15] Brunner A, Jöckel JA, Babst R (2008). The PFNA proximal femur nail in treatment of unstable proximal femur fractures--3 cases of postoperative perforation of the helical blade into the hip joint. J Orthop Trauma.

[16] Bousson VD, Adams J, Engelke K, Aout M, Cohen-Solal M, Bergot C, Haguenauer D, Goldberg D, Champion K, Aksouh R, Vicaut E, Laredo JD (2011). In vivo discrimination of hip fracture with quantitative computed tomography: results from the prospective European femur fracture study (EFFECT). J Bone Miner Res.

[17] Hwang JH, Garg AK, Oh JK, Oh CW, Lee SJ, Myung-Rae C, Kim MK, Kim H (2012). A biomechanical evaluation of proximal femoral nail antirotation with respect to helical blade position in femoral head: a cadaveric study. Indian J Orthop.

[18] Erhart S, Kammerlander C, El-Attal R, Schmoelz W (2012). Is augmentation a possible salvage procedure after lateral migration of the proximal femur nail antirotation?. Arch Orthop Trauma Surg.

[19] Kokoroghiannis C, Vasilakos D, Zisis K, Dimitriou G, Pappa E, Evangelopoulos D (2020). Is rotation the mode of failure in pertrochanteric fractures fixed with nails? Theoretical approach and illustrative cases. Eur J Orthop Surg Traumatol.

[20] Windolf M, Muths R, Braunstein V, Gueorguiev B, Hänni M, Schwieger K (2009). Quantification of cancellous bone-compaction due to DHS Blade insertion and influence upon cut-out resistance. Clin Biomech (Bristol, Avon).

[21] Sambandam SN, Chandrasekharan J, Mounasamy V, Mauffrey C (2016). Intertrochanteric fractures: a review of fixation methods. Eur J Orthop Surg Traumatol.

[22] Singh NK, Sharma V, Trikha V, Gamanagatti S, Roy A, Balawat AS, Aravindh P, Diwakar AR (2019). Is PFNA-II a better implant for stable intertrochanteric fractures in elderly population? A prospective randomized study. J Clin Orthop Trauma.

[23] Bonnaire F, Lein T, Fülling T, Bula P (2020). Reduced complication rates for unstable trochanteric fractures managed with third-generation nails: gamma 3 nail versus PFNA. Eur J Trauma Emerg Surg.

[24] Gok K, Inal S, Gok A, Gulbandilar E (2017). Comparison of effects of different screw materials in the triangle fixation of femoral neck fractures. J Mater Sci Mater Med.

[25] Lenich A, Bachmeier S, Prantl L, Nerlich M, Hammer J, Mayr E (2011). Is the rotation of the femoral head a potential initiation for cutting out? A theoretical and experimental approach. BMC Musculoskelet Disord.

[26] Erhart S, Schmoelz W, Blauth M, Lenich A (2011). Biomechanical effect of bone cement augmentation on rotational stability and pull-out strength of the proximal femur nail Antirotation™. Injury..

[27] Brunner A, Büttler M, Lehmann U, Frei HC, Kratter R, Di Lazzaro M (2016). What is the optimal salvage procedure for cut-out after surgical fixation of trochanteric fractures with the PFNA or TFN?: a multicentre study. Injury..

[28] Gray H, Standring S, Ellis H, Berkovitz BKB (2005). Gray’s anatomy: the anatomical basis of clinical practice.

[29] Ma JX, Kuang MJ, Fan ZR, Xing F, Zhao YL, Zhang LK, Chen HT, Han C, Ma XL (2017). Comparison of clinical outcomes with InterTan vs gamma nail or PFNA in the treatment of intertrochanteric fractures: a meta-analysis. Sci Rep.

[30] Yoon JY, Kim JW (2020). Treatment of proximal femur fracture with a newly designed nail: trochanteric fixation nail-advanced (TFNA). J Korean Fract Soc.

[31] Sermon A, Zderic I, Khatchadourian R, Scherrer S, Knobe M, Stoffel K, Gueorguiev B (2021). Bone cement augmentation of femoral nail head elements increases their cut-out resistance in poor bone quality- A biomechanical study. J Biomech.

